# Supplementary material for: Impact of chronic diseases on the periapical health of endodontically treated teeth: A systematic review and meta-analysis
Source: PLoS One. 2024 Feb 15;19(2):e0297020. doi: 10.1371/journal.pone.0297020 (PMC10868775; doi:10.1371/journal.pone.0297020)
Supplement: S2 Appendix — (DOCX) [file pone.0297020.s002.docx]

**Appendix S2 -** Database search strategy.

| **Database** | **Search** |
| --- | --- |
| **PubMed/Medline** | 1. (“Root Canal Obturation”[mh] OR “Root Canal Obturation” OR “Root Canal Obturations” OR “Endodontic Obturation” OR “Endodontic Obturations” OR “Dental Pulp Diseases”[mh] OR “Dental Pulp Diseases” OR “Dental Pulp Disease” OR “endodontic” OR “Endodontic Outcome” OR “endodontic outcomes” OR “endodontic treatment” OR “endodontic treatments” OR “root canal treatment” OR “endodontic therapy” OR “root canal therapy” OR “endodontics”) 2. (“systemic disease” OR “Chronic Disease” OR “Chronic Diseases” OR “Chronic Illness” OR “Chronic Illnesses” OR “systemic diseases” OR “diabetes” OR “Diabetes Mellitus”[mh] OR “Diabetes Mellitus” OR “HIV”[mh] OR “HIV” OR “Human Immunodeficiency Virus” OR “Human Immunodeficiency Viruses” OR “Human T Cell Lymphotropic Virus Type III” OR “Human T-Cell Lymphotropic Virus Type III” OR “Human T-Cell Leukemia Virus Type III” OR “Human T Cell Leukemia Virus Type III” OR “LAV-HTLV-III” OR “Lymphadenopathy-Associated Virus” OR “Lymphadenopathy Associated Virus” OR “Lymphadenopathy-Associated Viruses” OR “Human T Lymphotropic Virus Type III” OR “Human T-Lymphotropic Virus Type III” OR “AIDS Virus” OR “AIDS Viruses” OR “Acquired Immune Deficiency Syndrome Virus” OR “Acquired Immunodeficiency Syndrome Virus” OR “hypertension”[mh] OR “hypertension” OR “High Blood Pressure” OR “High Blood Pressures” OR “Cardiovascular Diseases”[mh] OR “Cardiovascular Diseases” OR “Cardiovascular Disease”) 3. #1 AND #2 |
| **SCOPUS** | TITLE-ABS-KEY(“Root Canal Obturation” OR “Root Canal Obturations” OR “Endodontic Obturation” OR “Endodontic Obturations” OR “Dental Pulp Diseases” OR “Dental Pulp Disease” OR “endodontic” OR “Endodontic Outcome” OR “endodontic outcomes” OR “endodontic treatment” OR “endodontic treatments” OR “root canal treatment” OR “endodontic therapy” OR “root canal therapy” OR “endodontics”) AND TITLE-ABS-KEY(“systemic disease” OR “Chronic Disease” OR “Chronic Diseases” OR “Chronic Illness” OR “Chronic Illnesses” OR “systemic diseases” OR “diabetes” OR “Diabetes Mellitus” OR “HIV” OR “Human Immunodeficiency Virus” OR “Human Immunodeficiency Viruses” OR “Human T Cell Lymphotropic Virus Type III” OR “Human T-Cell Lymphotropic Virus Type III” OR “Human T-Cell Leukemia Virus Type III” OR “Human T Cell Leukemia Virus Type III” OR “LAV-HTLV-III” OR “Lymphadenopathy-Associated Virus” OR “Lymphadenopathy Associated Virus” OR “Lymphadenopathy-Associated Viruses” OR “Human T Lymphotropic Virus Type III” OR “Human T-Lymphotropic Virus Type III” OR “AIDS Virus” OR “AIDS Viruses” OR “Acquired Immune Deficiency Syndrome Virus” OR “Acquired Immunodeficiency Syndrome Virus” OR “hypertension” OR “High Blood Pressure” OR “High Blood Pressures” OR “Cardiovascular Diseases” OR “Cardiovascular Disease”) |
| **WEB OF SCIENCE** | 1. TS=(“Root Canal Obturation” OR “Root Canal Obturations” OR “Endodontic Obturation” OR “Endodontic Obturations” OR “Dental Pulp Diseases” OR “Dental Pulp Disease” OR “endodontic” OR “Endodontic Outcome” OR “endodontic outcomes” OR “endodontic treatment” OR “endodontic treatments” OR “root canal treatment” OR “endodontic therapy” OR “root canal therapy” OR “endodontics”) 2. TS=(“systemic disease” OR “Chronic Disease” OR “Chronic Diseases” OR “Chronic Illness” OR “Chronic Illnesses” OR “systemic diseases” OR “diabetes” OR “Diabetes Mellitus” OR “HIV” OR “Human Immunodeficiency Virus” OR “Human Immunodeficiency Viruses” OR “Human T Cell Lymphotropic Virus Type III” OR “Human T-Cell Lymphotropic Virus Type III” OR “Human T-Cell Leukemia Virus Type III” OR “Human T Cell Leukemia Virus Type III” OR “LAV-HTLV-III” OR “Lymphadenopathy-Associated Virus” OR “Lymphadenopathy Associated Virus” OR “Lymphadenopathy-Associated Viruses” OR “Human T Lymphotropic Virus Type III” OR “Human T-Lymphotropic Virus Type III” OR “AIDS Virus” OR “AIDS Viruses” OR “Acquired Immune Deficiency Syndrome Virus” OR “Acquired Immunodeficiency Syndrome Virus” OR “hypertension” OR “High Blood Pressure” OR “High Blood Pressures” OR “Cardiovascular Diseases” OR “Cardiovascular Disease”) 3. #1 AND #2 |
| **COCHRANE LIBRARY** | (“Root Canal Obturation” OR “Root Canal Obturations” OR “Endodontic Obturation” OR “Endodontic Obturations” OR “Dental Pulp Diseases” OR “Dental Pulp Disease” OR “endodontic” OR “Endodontic Outcome” OR “endodontic outcomes” OR “endodontic treatment” OR “endodontic treatments” OR “root canal treatment” OR “endodontic therapy” OR “root canal therapy” OR “endodontics”) AND (“systemic disease” OR “Chronic Disease” OR “Chronic Diseases” OR “Chronic Illness” OR “Chronic Illnesses” OR “systemic diseases” OR “diabetes” OR “Diabetes Mellitus” OR “HIV” OR “Human Immunodeficiency Virus” OR “Human Immunodeficiency Viruses” OR “Human T Cell Lymphotropic Virus Type III” OR “Human T-Cell Lymphotropic Virus Type III” OR “Human T-Cell Leukemia Virus Type III” OR “Human T Cell Leukemia Virus Type III” OR “LAV-HTLV-III” OR “Lymphadenopathy-Associated Virus” OR “Lymphadenopathy Associated Virus” OR “Lymphadenopathy-Associated Viruses” OR “Human T Lymphotropic Virus Type III” OR “Human T-Lymphotropic Virus Type III” OR “AIDS Virus” OR “AIDS Viruses” OR “Acquired Immune Deficiency Syndrome Virus” OR “Acquired Immunodeficiency Syndrome Virus” OR “hypertension” OR “High Blood Pressure” OR “High Blood Pressures” OR “Cardiovascular Diseases” OR “Cardiovascular Disease”) |
| **LILACS** | ("Root Canal Obturation" OR "Root Canal Obturations" OR "Endodontic Obturation" OR "Endodontic Obturations" OR "Dental Pulp Diseases" OR "Dental Pulp Disease" OR "endodontic" OR "Endodontic Outcome" OR "endodontic outcomes" OR "endodontic treatment" OR "endodontic treatments" OR "root canal treatment" OR "endodontic therapy" OR "root canal therapy" OR "endodontics" OR "Obturação do canal radicular" OR "Obturação do canal radicular" OR "Obturação endodôntica" OR "Obturação endodôntica" OR "Doenças da polpa dentária" OR "Doença da polpa dentária" OR "endodôntico" OR "Resultado endodôntico" OR "Resultados endodônticos" OR "tratamento endodôntico" OR "tratamentos endodônticos" OR "tratamento de canal" OR "terapia endodôntica" OR "terapia de canal radicular" OR "endodontia" OR "Obturación del conducto radicular" OR "Obturaciones del conducto radicular" OR "Obturación endodóntica" OR "Obturaciones endodónticas" OR "Enfermedades de la pulpa dental" OR "Enfermedad de la pulpa dental" OR "endodóntico" OR "Resultado endodóntico" OR "resultados endodónticos" OR "tratamiento de endodoncia" OR "tratamientos de endodoncia" OR "tratamiento de conducto radicular" OR "terapia de endodoncia" OR "terapia de conducto radicular" OR "endodoncia") AND ("systemic disease" OR "Chronic Disease" OR "Chronic Diseases" OR "Chronic Illness" OR "Chronic Illnesses" OR "systemic diseases" OR "diabetes" OR "Diabetes Mellitus" OR "HIV" OR "Human Immunodeficiency Virus" OR "Human Immunodeficiency Viruses" OR "Human T Cell Lymphotropic Virus Type III" OR "Human T-Cell Lymphotropic Virus Type III" OR "Human T-Cell Leukemia Virus Type III" OR "Human T Cell Leukemia Virus Type III" OR "LAV-HTLV-III" OR "Lymphadenopathy-Associated Virus" OR "Lymphadenopathy Associated Virus" OR "Lymphadenopathy-Associated Viruses" OR "Human T Lymphotropic Virus Type III" OR "Human T-Lymphotropic Virus Type III" OR "AIDS Virus" OR "AIDS Viruses" OR "Acquired Immune Deficiency Syndrome Virus" OR "Acquired Immunodeficiency Syndrome Virus" OR "hypertension" OR "High Blood Pressure" OR "High Blood Pressures" OR "Cardiovascular Diseases" OR "Cardiovascular Disease" OR "Doença sistêmica" OR "Doença crônica" OR "Doenças crônicas" OR "Doença crônica" OR "Doenças crônicas" OR "doenças sistêmicas" OR "diabetes" OR "Diabetes Mellitus" OR "HIV" OR "Vírus da imunodeficiência humana" OR "Vírus da Imunodeficiência Humana" OR "Vírus Linfotrópico de Células T Humanas Tipo III" OR "Vírus Linfotrópico de Células T Humanas Tipo III" OR "Vírus da Leucemia de Células T Humanas Tipo III" OR "Vírus da Leucemia de Células T Humanas Tipo III" OR "LAV -HTLV-III" OR "Vírus associado a linfadenopatia" OR "Vírus associado a linfadenopatia" OR "Vírus associados a linfadenopatia" OR "Vírus linfotrópico T humano tipo III" OR "Vírus linfotrópico T humano tipo III" OR "Vírus da AIDS" OR "Vírus da AIDS" OR "Vírus da síndrome da imunodeficiência adquirida" OR "Vírus da síndrome da imunodeficiência adquirida" OR "hipertensão" OR "Pressão alta" OR "Pressão alta" OR "Doenças cardiovasculares" OR "Doença cardiovascular" OR "Enfermedad sistémica" OR "Enfermedad crónica" OR "Enfermedades crónicas" OR "Enfermedad crónica" OR "Enfermedades crónicas" OR "enfermedades sistémicas" OR "diabetes" OR "Diabetes Mellitus" OR "VIH" OR "Virus de inmunodeficiencia humana" OR "Virus de inmunodeficiencia humana" OR "Virus linfotrópico de células T humano tipo III" OR "Virus linfotrópico de células T humano tipo III" OR "Virus de leucemia de células T humano tipo III" OR "Virus de leucemia de células T humano tipo III" OR "LAV -HTLV-III" OR "Virus asociado a linfadenopatía" OR "Virus asociado a linfadenopatía" OR "Virus asociados a linfadenopatía" OR "Virus linfotrópico T humano tipo III" OR "Virus linfotrópico T humano tipo III" OR "Virus del SIDA" OR "Virus del SIDA" OR "Virus del síndrome de inmunodeficiencia adquirida" OR "Virus del síndrome de inmunodeficiencia adquirida" OR "hipertensión" OR "Presión arterial alta" OR "Presión arterial alta" OR "Enfermedades cardiovasculares" OR "Enfermedad cardiovascular") |
| **EMBASE** | ('root canal obturation':ab,ti,kw OR 'root canal obturations':ab,ti,kw OR 'endodontic obturation':ab,ti,kw OR 'endodontic obturations':ab,ti,kw OR 'dental pulp diseases':ab,ti,kw OR 'dental pulp disease':ab,ti,kw OR 'endodontic':ab,ti,kw OR 'endodontic outcome':ab,ti,kw OR 'endodontic outcomes':ab,ti,kw OR 'endodontic treatment':ab,ti,kw OR 'endodontic treatments':ab,ti,kw OR 'root canal treatment':ab,ti,kw OR 'endodontic therapy':ab,ti,kw OR 'root canal therapy':ab,ti,kw OR 'endodontics':ab,ti,kw) AND ('systemic disease':ab,ti,kw OR 'chronic disease':ab,ti,kw OR 'chronic diseases':ab,ti,kw OR 'chronic illness':ab,ti,kw OR 'chronic illnesses':ab,ti,kw OR 'systemic diseases':ab,ti,kw OR 'diabetes':ab,ti,kw OR 'diabetes mellitus':ab,ti,kw OR 'hiv':ab,ti,kw OR 'human immunodeficiency virus':ab,ti,kw OR 'human immunodeficiency viruses':ab,ti,kw OR 'human t cell lymphotropic virus type iii':ab,ti,kw OR 'human t-cell lymphotropic virus type iii':ab,ti,kw OR 'human t-cell leukemia virus type iii':ab,ti,kw OR 'human t cell leukemia virus type iii':ab,ti,kw OR 'lav-htlv-iii':ab,ti,kw OR 'lymphadenopathy-associated virus':ab,ti,kw OR 'lymphadenopathy associated virus':ab,ti,kw OR 'lymphadenopathy-associated viruses':ab,ti,kw OR 'human t lymphotropic virus type iii':ab,ti,kw OR 'human t-lymphotropic virus type iii':ab,ti,kw OR 'aids virus':ab,ti,kw OR 'aids viruses':ab,ti,kw OR 'acquired immune deficiency syndrome virus':ab,ti,kw OR 'acquired immunodeficiency syndrome virus':ab,ti,kw OR 'hypertension':ab,ti,kw OR 'high blood pressure':ab,ti,kw OR 'high blood pressures':ab,ti,kw OR 'cardiovascular diseases':ab,ti,kw OR 'cardiovascular disease':ab,ti,kw) |
| **Cinahl** | (“Root Canal Obturation” OR “Root Canal Obturations” OR “Endodontic Obturation” OR “Endodontic Obturations” OR “Dental Pulp Diseases” OR “Dental Pulp Disease” OR “endodontic” OR “Endodontic Outcome” OR “endodontic outcomes” OR “endodontic treatment” OR “endodontic treatments” OR “root canal treatment” OR “endodontic therapy” OR “root canal therapy” OR “endodontics”) AND (“systemic disease” OR “Chronic Disease” OR “Chronic Diseases” OR “Chronic Illness” OR “Chronic Illnesses” OR “systemic diseases” OR “diabetes” OR “Diabetes Mellitus” OR “HIV” OR “Human Immunodeficiency Virus” OR “Human Immunodeficiency Viruses” OR “Human T Cell Lymphotropic Virus Type III” OR “Human T-Cell Lymphotropic Virus Type III” OR “Human T-Cell Leukemia Virus Type III” OR “Human T Cell Leukemia Virus Type III” OR “LAV-HTLV-III” OR “Lymphadenopathy-Associated Virus” OR “Lymphadenopathy Associated Virus” OR “Lymphadenopathy-Associated Viruses” OR “Human T Lymphotropic Virus Type III” OR “Human T-Lymphotropic Virus Type III” OR “AIDS Virus” OR “AIDS Viruses” OR “Acquired Immune Deficiency Syndrome Virus” OR “Acquired Immunodeficiency Syndrome Virus” OR “hypertension” OR “High Blood Pressure” OR “High Blood Pressures” OR “Cardiovascular Diseases” OR “Cardiovascular Disease”) |
| **Google Scholar** | endodontic AND “systemic disease” |
| **Open grey** | endodontic AND “systemic disease” |
| **Proquest** | noft(“Root Canal Obturation” OR “Root Canal Obturations” OR “Endodontic Obturation” OR “Endodontic Obturations” OR “Dental Pulp Diseases” OR “Dental Pulp Disease” OR “endodontic” OR “Endodontic Outcome” OR “endodontic outcomes” OR “endodontic treatment” OR “endodontic treatments” OR “root canal treatment” OR “endodontic therapy” OR “root canal therapy” OR “endodontics”) AND noft(“systemic disease” OR “Chronic Disease” OR “Chronic Diseases” OR “Chronic Illness” OR “Chronic Illnesses” OR “systemic diseases” OR “diabetes” OR “Diabetes Mellitus” OR “HIV” OR “Human Immunodeficiency Virus” OR “Human Immunodeficiency Viruses” OR “Human T Cell Lymphotropic Virus Type III” OR “Human T-Cell Lymphotropic Virus Type III” OR “Human T-Cell Leukemia Virus Type III” OR “Human T Cell Leukemia Virus Type III” OR “LAV-HTLV-III” OR “Lymphadenopathy-Associated Virus” OR “Lymphadenopathy Associated Virus” OR “Lymphadenopathy-Associated Viruses” OR “Human T Lymphotropic Virus Type III” OR “Human T-Lymphotropic Virus Type III” OR “AIDS Virus” OR “AIDS Viruses” OR “Acquired Immune Deficiency Syndrome Virus” OR “Acquired Immunodeficiency Syndrome Virus” OR “hypertension” OR “High Blood Pressure” OR “High Blood Pressures” OR “Cardiovascular Diseases” OR “Cardiovascular Disease”) |
| **MedvRix** | endodontic AND “systemic disease” |
