# Supplementary material for: Impact of chronic diseases on the periapical health of endodontically treated teeth: A systematic review and meta-analysis
Source: PLoS One. 2024 Feb 15;19(2):e0297020. doi: 10.1371/journal.pone.0297020 (PMC10868775; doi:10.1371/journal.pone.0297020)
Supplement: S3 Appendix — (DOCX) [file pone.0297020.s003.docx]

**Appendix S3 –** Excluded articles (n = 20).

| **Author, Year** | **Reason for Exclusion** |
| --- | --- |
| Affrin,2016^1^ | 4 |
| Juncar et al., 2019^2^ | 3 |
| Keerthana et al. 2020^3^ | 4 |
| Sanchez-Dominguez et al. 2015^4^ | 3 |
| Segura Egea et al., 2012^5^ | 5 |
| Segura Egea et al., 2010^6^ | 3 |
| Segura Egea et al., 2015^7^ | 5 |
| De Brito et al., 2015^8^ | 4 |
| Gomes-Filho et al., 2014^9^ | 5 |
| Gomes et al., 2016^10^ | 2 |
| Ilguy, et al., 2007^11^ | 2 |
| Jahreis et al., 2019^12^ | 3 |
| Katz et al., 2020^13^ | 3 |
| Mindiola et al., 2006^14^ | 4 |
| Ng et al., 2011^15^ | 4 |
| Quezada et al., 2018^16^ | 5 |
| Gonzalez-Navarro et al., 2020^17^ | 2 |
| Yip et al., 2021^18^ | 2 |
| Suchina et al., 2006^19^ | 3 |
| Wang et al., 2011^20^ | 4 |

1. Excluded studies where patients had deciduous or mixed dentition.
2. Excluded studies where patients had acute illness or were healthy, or endodontic treatment was not performed.
3. Excluded studies where there was no comparison with healthy individuals.
4. Excluded studies that did not have an appropriate evaluation method or lacked the outcome of interest – Healing confirmed by clinical or radiographic method. Studies on the survival of endodontically treated teeth (extraction/non-extraction).
5. Excluded studies such as editorials, case reports, case series, expert opinions, guidelines, and reviews.

**REFERENCE**

1. Affrin. Diabetes mellitus associated with oral disease – A retrospective study. *J Pharm Sci Res*. 2016;8(8):914-915.

2. Juncar RJ, Precup AI, Juncar M. Odontogenic inflammatory lesions in patients with type 2 diabetes mellitus: A prospective study of 128 cases. *Nigerian journal of clinical practice*. 23(3):298-303.

3. Keerthana R, Nasim I, Chaudhary M. Association between failed root canal treatment and systemic diseases. *Journal of Complementary Medicine Research*. 2020;11(4):46-51.

4. Sanchez-Dominguez B, Lopez-Lopez J, Jane-Salas E, Castellanos-Cosano L, Velasco-Ortega E, Segura-Egea JJ. Glycated Hemoglobin Levels and Prevalence of Apical Periodontitis in Type 2 Diabetic Patients. *J Endod*. 41(5):601-606.

5. Segura-Egea JJ, Castellanos-Cosano L, Machuca G, et al. Diabetes mellitus, periapical inflammation and endodontic treatment outcome. *Med Oral Patol Oral Cir Bucal*. 17(2):E356-E361.

6. Segura-Egea JJ, Jimenez-Moreno E, Calvo-Monroy C, et al. Hypertension and Dental Periapical Condition. *J Endod*. 36(11):1800-1804.

7. Segura-Egea JJ, Martin-Gonzalez J, Castellanos-Cosano L. Endodontic medicine: connections between apical periodontitis and systemic diseases. *Int Endod J*. 48(10):933-951.

8. de Brito LC, Teles FR, Teles RP, Nogueira PM, Vieira LQ, Ribeiro Sobrinho AP. Immunological profile of periapical endodontic infections from HIV- and HIV+ patients. *Int Endod J*. 48(6):533-41.

9. Gomes-Filho JE, Martins CM, Sivieri-Araujo G, et al. Influence of hypertension on oral infections and endodontic treatment. *Dent Press Endod*. 2014;4(1):15-20.

10. Gomes MS, Hugo FN, Hilgert JB, et al. Apical periodontitis and incident cardiovascular events in the Baltimore Longitudinal Study of Ageing. *Int Endod J*. 49(4):334-42.

11. Ilgüy M, Ilgüy D, Bayirli G. Dental lesions in adult diabetic patients. *The New York state dental journal*. 73(1):58-60.

12. Jahreis M, Soliman S, Schubert A, et al. Outcome of non-surgical root canal treatment related to periodontitis and chronic disease medication among adults in age group of 60 years or more. *Gerodontology*. 36(3):267-275.

13. Katz J, Rotstein I. Prevalence of Periapical Lesions in Patients with Osteoporosis. *J Endod*. 47(2):234-238.

14. Mindiola MJ, Mickel AK, Sami C, Jones JJ, Lalumandier JA, Nelson SS. Endodontic treatment in an American Indian population: a 10-year retrospective study. *J Endod*. Sep 2006;32(9):828-32. doi:10.1016/j.joen.2006.03.007

15. Ng YL, Mann V, Gulabivala K. A prospective study of the factors affecting outcomes of non-surgical root canal treatment: part 2: tooth survival. *Int Endod J*. Jul 2011;44(7):610-25. doi:10.1111/j.1365-2591.2011.01873.x

16. Quezada García MA, Palma Eyzaguirre AM. Relación bidireccional entre diabetes mellitus y periodontitis apical. *ARS med (Santiago, En línea)*. 2018;43(3):67-76.

17. Gonzalez-Navarro B, Segura-Egea JJ, Estrugo-Devesa A, et al. Relationship between Apical Periodontitis and Metabolic Syndrome and Cardiovascular Events: A Cross-Sectional Study. *Journal of clinical medicine*. 9(10)

18. Yip N, Liu C, Wu D, Fouad AF. The association of apical periodontitis and type 2 diabetes mellitus: A large hospital network cross-sectional case-controlled study. *Journal of the American Dental Association (1939)*. 152(6):434-443.

19. Suchina JA, Levine D, Flaitz CM, Nichols CM, Hicks MJ. Retrospective clinical and radiologic evaluation of nonsurgical endodontic treatment in human immunodeficiency virus (HIV) infection. *The journal of contemporary dental practice*. 7(1):1-8.

20. Wang CH, Chueh LH, Chen SC, Feng YC, Hsiao CK, Chiang CP. Impact of diabetes mellitus, hypertension, and coronary artery disease on tooth extraction after nonsurgical endodontic treatment. *J Endod*. Jan 2011;37(1):1-5. doi:10.1016/j.joen.2010.08.054
