# Supplementary material for: Impact of chronic diseases on the periapical health of endodontically treated teeth: A systematic review and meta-analysis
Source: PLoS One. 2024 Feb 15;19(2):e0297020. doi: 10.1371/journal.pone.0297020 (PMC10868775; doi:10.1371/journal.pone.0297020)
Supplement: Attachment — Submitted filename: Revision notes.docx [file pone.0297020.s004.docx]

**POINT-BY-POINT RESPONSE TO REVIEWERS**

Dear Sir,

I would like to express my gratitude to the reviewers for their valuable feedback regarding our article. We have taken your comments into careful consideration and made all the necessary corrections as well as addressed the questions raised.

Thank you once again for your time and effort in reviewing our work. We greatly appreciate your input and are confident that the improvements made will enhance the overall quality of our article.

Reply to Reviewer #1

The article is well written. A thorough research and analyses has been systematically done.
The topic is important and relevant.
The importance of study is properly highlighted and the reasons for conducting it are stated clearly.
The results are presented clearly.
Important findings and results are discussed.
Conclusion is in accordance with data analysis and results.

Overall the Introduction, Methodology and Discussion have covered all aspects related to the study. However shedding a little light on any future recommendations would have made it great.

**ANSWER:** We appreciate the comments made and have taken the suggestion into account. Future recommendations have been added to the text in the discussion section.

Reply to Reviewer #2:

1. Conclusions are not alligned with the objectives of the study

**ANSWER:** We appreciate the feedback and have taken the suggestion into account. Accordingly, the conclusion has been rephrased.

2. Conclusion is not specific and clear

**ANSWER:** We appreciate the feedback and have taken the suggestion into account. Accordingly, the conclusion has been rephrased.

3. Inclusion criteria does not mention the inclusion of studies with PAI index only. It generally mentions that the studies with outcome measured with clinical and radiographic outcome were included

**ANSWER:** Thank you for the feedback. We accepted for inclusion any study that used a standardized and validated metric, such as the PAI index. Additionally, other standardized and validated forms were equally considered for inclusion. The decision to employ the PAI index in the meta-analysis was motivated by the fact that the majority of included studies adopted this metric. This clarification has been made in the text.

4. No data with respect to PAI index in the 23 studies is mentioned, since discussion is revolving around it.

**ANSWER:** Thank you for the feedback. We accepted for inclusion any study that used a standardized and validated metric, such as the PAI index. Additionally, other standardized and validated forms were equally considered for inclusion. The decision to employ the PAI index in the meta-analysis was motivated by the fact that the majority of included studies adopted this metric. This clarification has been made in the text.

5. The detail under the heading of " results of individual studies" is too lengthy, the information should be crisp and concise and summarized briefly into tabular form.

**ANSWER:** The suggestion was accepted, and the section on individual study results was rewritten. The information for each individual study is provided in Table 1.

6. Interprettation of MetaAnalysis specific for diabetic patients is not clearly described with respect to PAI in results.

**ANSWER:** Thank you for the feedback. The interpretation of the meta-analysis has been rewritten to make it clearer.

7. The study is not registered in PROSPERO uptill now

**ANSWER:** We appreciate the comment. The registration was completed at the beginning of the review; however, the verb tense in the text was incorrect. This has been rectified. The protocol number was not included in the text to allow for a blinded peer review.
